# Effects of Blood Flow Restricted Low-Intensity Concentric or Eccentric Training on Muscle Size and Strength

**DOI:** 10.1371/journal.pone.0052843

**Published:** 2012-12-31

**Authors:** Tomohiro Yasuda, Jeremy P. Loenneke, Robert S. Thiebaud, Takashi Abe

**Affiliations:** 1 Department of Ischemic Circulatory Physiology, University of Tokyo, Tokyo, Japan; 2 Department of Health and Exercise Science, University of Oklahoma, Norman, Oklahoma, United States of America; 3 Department of Health, Exercise Science, & Recreation Management, University of Mississippi, Oxford, Mississippi, United States of America; University of Zaragoza, Spain

## Abstract

We investigated the acute and chronic effects of low-intensity concentric or eccentric resistance training with blood flow restriction (BFR) on muscle size and strength. Ten young men performed 30% of concentric one repetition maximal dumbbell curl exercise (four sets, total 75 reps) 3 days/week for 6 weeks. One arm was randomly chosen for concentric BFR (CON-BFR) exercise only and the other arm performed eccentric BFR (ECC-BFR) exercise only at the same exercise load. During the exercise session, iEMG for biceps brachii muscles increased progressively during CON-BFR, which was greater (p<0.05) than that of the ECC-BFR. Immediately after the exercise, muscle thickness (MTH) of the elbow flexors acutely increased (p<0.01) with both CON-BFR and ECC-BFR, but was greater with CON-BFR (11.7%) (p<0.01) than ECC-BFR (3.9%) at 10-cm above the elbow joint. Following 6-weeks of training, MRI-measured muscle cross-sectional area (CSA) at 10-cm position and mid-upper arm (12.0% and 10.6%, respectively) as well as muscle volume (12.5%) of the elbow flexors were increased (p<0.01) with CON-BFR. Increases in muscle CSA and volume were lower in ECC-BFR (5.1%, 0.8% and 2.9%, respectively) than in the CON-BFR and only muscle CSA at 10-cm position increased significantly (p<0.05) after the training. Maximal voluntary isometric strength of elbow flexors was increased (p<0.05) in CON-BFR (8.6%), but not in ECC (3.8%). These results suggest that CON-BFR training leads to pronounced acute changes in muscle size, an index of muscle cell swelling, the response to which may be an important factor for promoting muscle hypertrophy with BFR resistance training.

## Introduction

In the past decade, several studies have reported that low-intensity resistance training (20–30% 1RM) combined with blood flow restriction (BFR) elicits similar muscle hypertrophy as traditional high-intensity resistance training (>70% 1-RM) regardless of age [1], [2], [3], [4]. This relatively new training technique may be an alternative training method to improve muscle size and strength in healthy individuals or older adults and patients with health risks related with high-intensity resistance training. Recent studies demonstrated that a single bout of low-intensity resistance exercise with BFR stimulates the anabolic cell signaling mammalian target of rapamycin (mTOR) pathway, resulting in increased muscle protein synthesis within 3 hours after exercise [5], [6], [7]. Similarly, the downregulation of proteolytic transcripts have been observed at 8 hours following low-intensity resistance exercise with BFR [8]. The physiological mechanisms that promote muscle growth associated with low-intensity exercise combined with BFR are poorly understood, although several possibilities exist [9], [10], [11].

Acute cell swelling has been shown to stimulate protein synthesis and suppress proteolysis [12], [13]. A previous study reported that increased leg circumference, an index of muscle swelling, was more pronounced in BFR than in non-BFR immediately after low-intensity knee extension exercise [6]. Furthermore, following a single bout of low-intensity BFR bench press exercise, acute changes in muscle size were observed in both the blood flow restricted triceps muscle as well as the blood flow non-restricted chest muscle, and both the triceps and chest muscles increased muscle cross-sectional area following BFR bench press training [14]. Therefore, it appears that BFR training-induced muscle cell swelling may contribute significantly to the anabolic benefits of BFR [15], [16].

A recent study demonstrated that acute changes in muscle cross-sectional area (CSA) of the vastus lateralis at mid-thigh tended to be higher with concentric BFR (ES = 0.13) compared to eccentric BFR (ES = 0.07) 24 hours after low-intensity knee extension exercise [17]. This taken together with the aforementioned swelling hypothesis [15] suggests that concentric muscle actions may play an important role in promoting the muscle hypertrophy observed following low-intensity resistance training with BFR. In contrast, it is well established that most studies investigating high-intensity resistance training demonstrated that eccentric training is more effective than repetition matched concentric training for muscle hypertrophy [18], [19], [20]. Therefore, the mechanisms underlying the muscle hypertrophy may differ between low-intensity resistance training with BFR and high-intensity resistance training. We hypothesized that muscle hypertrophy and strength gain would be higher with concentric BFR (CON-BFR) compared to eccentric BFR (ECC-BFR) following repetition matched low-intensity resistance training. Thus, the purpose of the present study was to investigate the acute and chronic effects of low-intensity blood flow restricted concentric or eccentric resistance training on muscle size and strength.

## Methods

### Subjects

Ten healthy young men (mean age, 22 [SD 2] yrs; standing height, 170.7 [SD 5.3] cm; body mass, 61.8 [SD 6.6] kg; upper-arm length measured as the distance between the lateral epicondyle of the humerus and the acromial process of the radius, 32.2 [SD 1.1] cm; systolic arterial pressure, 108 [SD 11] mmHg; diastolic arterial pressure 59 [SD 8] mmHg) volunteered for the study. None of the subjects had participated in resistance-type training for a minimum of 1 year prior to the start of the study. Each subject was informed of the risks associated with the training, measurements and the purpose of the study, which conformed to the Declaration of Helsinki and was approved by the Ethics Committee for Human Experiments, University of Tokyo. Written informed consent was obtained from each subject prior to participation.

### Training Protocol

Subjects performed a supervised arm curl exercise 3 days/week for 6 weeks. One week before the training, all subjects performed practice sessions for the concentric one-repetition maximum (1-RM) test and maximum isometric strength measurement. In addition, subjects were familiarized to the BFR stimulus. Three or four days before the training, the concentric 1-RM for each arm was determined. Subjects performed 5–6 unilateral biceps curls with a low load (approximately 30–40% predicted 1-RM) as a warm-up and to familiarize subjects with the biceps curl exercise. After a warm-up period, the intensity was set at about 80% of predicted 1-RM. Following each successful lift, the intensity was increased by about 5% until the subject failed to lift through the entire range of motion. A test was considered valid only when the subject used proper form and completed the entire lift in a controlled manner without assistance. On average, five trials were required to complete a 1-RM test (2–3 min rest between each attempt). Training intensity and volume were set at 30% of concentric 1-RM and 75 repetitions (30 reps and the next 3 sets each consisting of 15 reps, with 30 sec of rest between sets) for each arm respectively. This protocol is typical of submaximal BFR studies [14], [21]. One arm was randomly chosen to perform concentric exercise, while the other arm performed eccentric exercise at the same exercise load. In a randomized order, either concentric or eccentric exercise was performed first followed by the other exercise completed on the same day. During these protocols, subjects performed their respective action with a cadence of 1.5-sec for concentric (shortening) or 1.5-sec for eccentric (lengthening) using a metronome, and the investigators manually performed the opposite muscle action. Elbow joint range of motion (ROM) during CON-BFR exercise was completed from full extension to full flexion, whereas ECC-BFR was completed from full flexion to full extension. The subjects were instructed to refrain from ingesting alcohol and caffeine for 24 hour prior to pre- and post-training measurements.

### Blood Flow Restriction

During the training sessions, subjects wore a specially designed elastic pressure cuff (30 mm wide, KAATSU Master, Sato Sports Plaza, Tokyo, Japan) around the most proximal portion of the upper arm. On the first day of training, the cuff was set at 100 mmHg. The pressure was increased by 10 mmHg at each subsequent training session until a pressure of 160 mmHg was reached. The restriction pressure was selected in accordance with previous studies [21], [22]. The pressure intensity was the same between concentric and eccentric exercises at every session. Immediately after the exercise bout, the pressure cuff was quickly removed. The amount of time under moderate blood flow restriction was approximately 5 min.

### Measurements Schedule

Subject testing took place before the start of the study (pre) and 3–4 days after (post) the 6-week training period. The MRI measurement was obtained between 13∶00 and 18∶00 hours. The MVC measurement was determined on the same day or the following day after the MRI measurement. All measurements were balanced for the time of day.

### Maximum Isometric Strength Measurement

Maximum voluntary isometric contraction (MVC) of the elbow flexors was measured twice by a dynamometer (Taiyo kogyo Co., Tokyo, Japan). Each subject was comfortably seated on an adjustable chair, with the arm positioned on a stable table at chest level with the elbow bent at an angle of 90° (0° at full extension). The upper arm was maintained in the horizontal plane (at 90°), while the wrist was fixed at the end of the dynamometer lever arm in a position of supination. The elbow flexion force was measured with a transducer while the subject’s performed two trials separated by a 60-sec rest interval. If MVC torque for the first two MVCs varied by more than 5%, up to two additional MVCs were performed with 60-sec rest between trials [23], [24]. Subjects were instructed to perform an MVC as quickly as possible during a period of about 5-sec. The recorded value for the MVC was taken as the highest and most stable ∼3 sec of the 5-sec contraction. The highest MVC value was used for data analysis. The coefficient variation (CV) for this measurement from test to retest was 1.3%. The intraclass correlation coefficient (ICC) of the measurements was 0.97.

### MRI-measured Muscle CSA and Volume

Muscle CSA was obtained using a magnetic resonance imaging (MRI) scanner (0.2-T Open MRI, Hitachi, Tokyo, Japan). A T-1 weighted, spin-echo, axial plane sequence was performed with a 500-msec repetition time and a 23-msec echo time. Subjects rested quietly in the magnet bore in a supine position, with their arms extended along their trunk. Continuous transverse images with 10-mm slice thickness were obtained from the both upper arms of the body. All MRI data were transferred to a personal computer for analysis using specially designed image analysis software (sliceOmatic, Tomovision Inc., QC, Canada). For each slice, skeletal muscle tissue cross-sectional area (CSA) was digitized, and the muscle tissue volume (cm^3^) per slice was calculated by multiplying muscle tissue area (cm^2^) by slice thickness (cm). Volume of elbow flexor (biceps brachii and brachialis) muscles was analyzed as the sum of the slices of muscle from 4 to 23 cm from the elbow joint. The CV of this measurement was less than 1.0% [14].

### Electromyography (EMG)

The skin was shaved, abraded with a skin preparation gel (Skinpure, Nihon Kohden, Japan), and cleaned with alcohol wipes. During the experiment, skin impedance was less than 2kΩ. The ground electrode was positioned on the lateral epicondyle. Bipolar (2-cm center-to-center) surface EMG (sEMG) electrodes (Ag/AgCl; Vitrode F; Nihon Kohden; Tokyo, Japan) were placed over the muscle belly (mid-portion) along the longitudinal axis of the testing upper arm [23], [24]. EMG signals were recorded and collected on a personal computer (T7300 Macintosh, Apple, Japan) for subsequent analysis. All EMG signals were digitized at a sampling rate of 1024 Hz with a bandwidth of 0 Hz to 500 kHz (AB 6216; Nihon Kohden; Tokyo, Japan). To determine integrated EMG (iEMG), signals were fully rectified and integrated (Power Lab Chart 5 software, ADInstruments, Japan). During the experimental session, sEMG was recorded continuously and each iEMG value was divided into groups of 5 successive repetitions. The average for each group of 5 repetitions was represented as a single data point for statistical analysis [23], [25]. This measurement was completed at 16^th^ or 17^th^ training session and both CON-BFR and ECC-BFR were performed at same day for each subject. The CV for this measurement from test to retest was 5.7%.

### Ultrasound-measured Muscle Thickness

Muscle thickness of the elbow flexors was measured using B-mode ultrasound (Acuson Sequoia 512, Siemens, Tokyo, Japan) at 10 cm above the elbow joint and at mid-upper arm. Briefly, the measurements were carried out while the subjects stood with their elbows extended and relaxed. A 10.0 MHz scanning head (5.5 cm length probe) was placed on the skin perpendicular to the tissue interface. The scanning head was coated with a water-soluble transmission gel to provide acoustic contact without depressing the dermal surface. The subcutaneous adipose tissue-muscle interface and the muscle-bone interface were identified from the ultrasonic image. The perpendicular distance from the adipose tissue-muscle interface to the muscle-bone interface was taken as muscle thickness (MTH). Ink markers on the elbow flexors were used to ensure similar positioning over repeated MTH measurement. The CV of this measurement from test to retest was 1.0% for 10 cm above the elbow joint and 1.4% for mid-upper arm. The ICCs of the measurements were 0.94 and 0.96, respectively. The same investigator (TY) made all the ultrasound measurements. The MTH was recorded before and immediately after the exercise bout. This measurement was completed at every week (1^st^–6^th^ week) of training period, and the average of 6 times for “before” or “immediately after” was represented as a single data point for statistical analysis, respectively.

### Ratings of Perceived Exertion

Ratings of perceived exertion (RPE), which is a scale (6–20) to measure subjective feelings of exertion and fatigue, was recorded immediately after the last set of every exercise [26].

### Statistical Analyses

Results are mean ± standard deviation (SD). Statistical analysis was performed by a two-way analysis of variance (ANOVA) with repeated measures [trials (CON-BFR vs. ECC-BFR)×time (pre vs. post)]. Post hoc testing was performed using Tukey’s test when appropriate. Percent changes from pre were also compared between groups using Tukey’s test. Statistical significance was set at p<0.05. Pre/post effect sizes (ESs, Cohen’s d) in MTH, muscle CSA and MVC were calculated with the following formula: ([posttraining mean – pretraining mean]/pretraining SD; d = 0.2 is small effect, d = 0.5 is a moderate effect, and d = 0.8 is a large effect) [27].

## Results

None of subjects had high systolic (≥130 mmHg) or diastolic (≥85 mmHg) blood pressure. Before training, there were no differences between CON-BFR and ECC-BFR for muscle CSA at 10-cm above the elbow joint and mid-upper arm, elbow flexor muscle volume (Table 1), MVC (37.9±4.7 and 37.6±4.9 Nm, respectively), and 1-RM strength (10.6±1.1 and 10.5±1.5 kg, respectively). There was no change in body weight following the training.

**Table 1:** Changes in cross-sectional area and volume of elbow flexors muscles.

	CON-BFR				ECC-BFR			
Muscle	Pre	Post	%Δ	ES	Pre	Post	%Δ	ES
CSA at mid-upper arm (cm^2^)	8.9 (0.8)	9.8 (0.7)*	10.0^#^	1.1^c^	9.9 (0.9)	9.7 (1.2)	−2.0	−0.2
CSA at 10 cm above theelbow joint (cm^2^)	14.7 (1.0)	16.4 (1.4)**	12.0##	1.7^c^	15.4 (1.7)	16.2 (2.0)*	5.1	0.5^b^
Volume (cm^3^)	219 (16)	246 (17)**	12.5##	1.6^c^	228 (21)	235 (23)	2.9	0.3^a^

Values are means ± SD. ES, effect size. Significant differences between CON-BFR training and ECC-BFR training:

## p<0.01. Significant differences between pre- and post-training:

** p<0.01,

* p<0.05.

a = small ES,

b = moderate ES,

c = large ES.

### Acute Effect of CON-BFR and ECC-BFR

**Figure 1:**
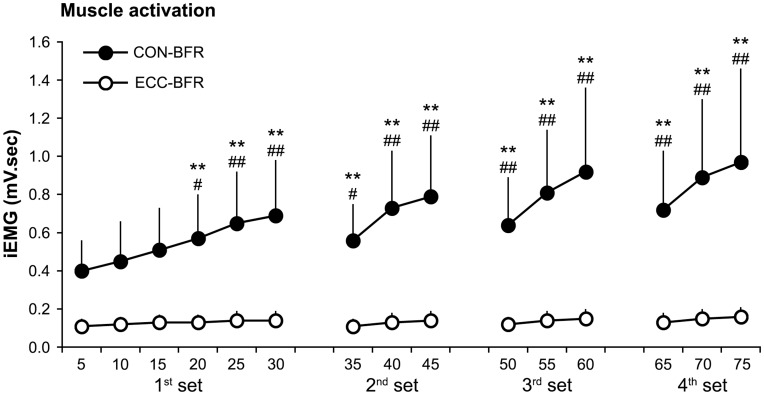
Muscle activation of biceps brachii muscles during concentric or eccentric BFR exercise for each set performed. Average for each 5-repetitions was represented as a single data point. Values are means and SD. **Different from first 5-reps, P<0.01. ##Different from ECC-BFR, P<0.01. #Different from ECC-BFR, P<0.05.

**Figure 2:**
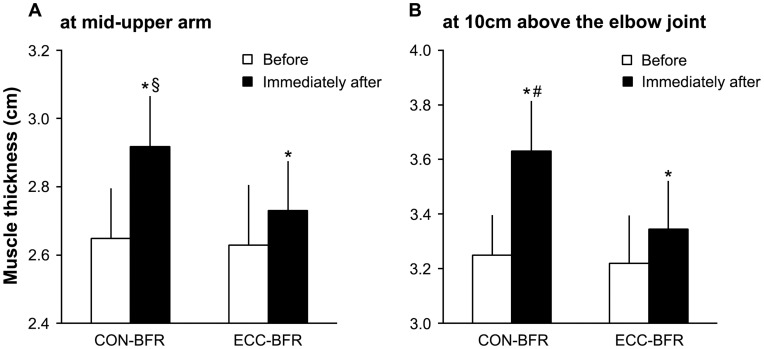
Muscle thickness (MTH) of the elbow flexors before and immediately after exercise session. Values are means and SD. *Different from before, P<0.05. #Different from ECC-BFR, P<0.05. §Different from ECC-BFR, P = 0.07.

During the exercise session, iEMG increased (p<0.05) progressively during CON-BFR and was greater (p<0.05) with CON-BFR than ECC-BFR from the 1^st^ to last set (Figure 1). Mean iEMG during exercise was higher (p<0.01) with CON-BFR (0.69±0.29 mV sec) than ECC-BFR (0.13±0.04 mV sec). Immediately after the exercise session, mean MTH increased (p<0.01) in both CON-BFR and ECC-BFR and was greater (p<0.01) with the CON-BFR (11.7%) compared to the ECC-BFR (3.9%) at 10 cm above the elbow joint. Also, changes in MTH at the mid-upper arm with CON-BFR (10.2%) tended to be greater (p = 0.07) than ECC-BFR (4.0%). ESs for the differences in MTH between before and immediately were large for CON-BFR (2.59 for at 10 cm above the elbow joint and 1.83 for at mid-upper arm), but were moderate for ECC-BFR (0.71 for at 10 cm above the elbow joint and 0.57 for at mid-upper arm) (Figure 2A&B). RPE was greater (p<0.01) with CON-BFR (16.4±1.4) than ECC-BFR (11.6±1.3).

### Chronic Effect of CON-BFR and ECC-BFR

**Figure 3:**
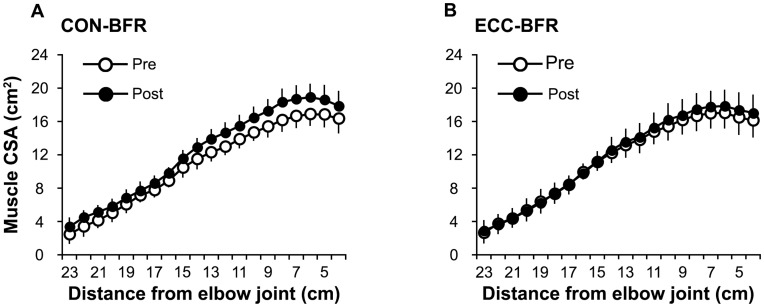
Distribution of muscle cross-sectional area (CSA) in elbow flexors pre- and post-training period. Values are means and SD.

**Figure 4:**
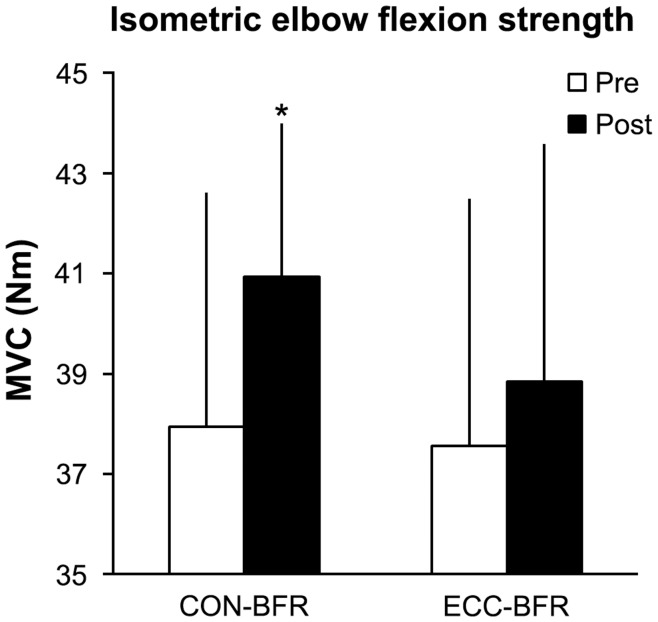
Maximum isometric strength (MVC) of the elbow flexors pre- and post- training period. Values are means and SD. *Different from pre-training, P<0.05.

ESs for the differences in muscle distribution between pre- and post-training were large (0.81–1.70 for 4–17 cm and 21–23 cm above the elbow joint) or moderate for CON-BFR (0.68–0.73 for 18–20 cm distance from elbow joint) (Figure 3A), but were small for ECC-BFR (0.24–0.46 for 4–11 cm and 13–14 cm above the elbow joint) (Figure 3B). Muscle CSA at 10 cm above the elbow joint and mid-upper arm (12.0% and 10.0%, respectively) and muscle volume (12.5%) were increased (p<0.01) in CON-BFR, while ECC-BFR only increased (p<0.05) muscle CSA at 10 cm above the elbow joint (5.1%). There were no changes in muscle volume (2.9%) or muscle CSA at the mid upper arm (−2.0%) with ECC-BFR (Table 1). Isometric MVC strength was increased (p<0.05) in CON-BFR (8.6%), but not in ECC-BFR (3.8%). ESs for the difference in MVC between pre- and post-training was moderate for CON-BFR (0.64), but was small for ECC-BFR (0.26) (Figure 4).

## Discussion

This study demonstrated that muscle hypertrophy and strength gain from BFR in combination with resistance training mostly occurs from the concentric portion of the lift (muscle volume 12.5% and muscle strength 8.6%) but not the eccentric portion (muscle volume 2.9% and muscle strength 3.8%). Therefore, it could be stated that the concentric phase of dynamic BFR resistance exercise appears to be the most important for causing acute increased muscle size (cell swelling) and appears to stimulate a greater muscle hypertrophic response than the eccentric phase.

With respect to acute MTH responses, CON-BFR exercise resulted in greater acute MTH compared to ECC-BFR exercise. Pilot biopsy data from our laboratory suggest that low-intensity knee extension exercise with BFR results in decreased plasma volume and an acute increase in muscle fiber CSA of the BFR muscle (Abe et al., 2012). Therefore, the higher muscle cell swelling may be observed following CON-BFR exercise. It is known that acute cell swelling due to osmotic water shifts into the cell simulate anabolic processes, both through increases in protein synthesis and decrease in proteolysis [12], [28], [29], [30]. Thus, the CON-BFR exercise-induced enhancement of muscle protein metabolism may be the basis for the increases in muscle size observed.

During BFR exercise, muscle activation increased progressively only when CON-BFR exercise was performed. In general, muscle activations are higher during concentric actions than during eccentric actions when submaximal exercise is performed at the same absolute load [31]. Additionally, an increase in lactate production has been found to be more pronounced with concentric actions compared with eccentric actions [32], [33]. In previously reported BFR studies, the greater muscle activation during low-intensity BFR resistance exercise was hypothesized to occur in order to compensate for a deficit in force development secondary to changes in energy supply; resulting from a decreased oxygen availability to the muscle and an accumulation of metabolites [22], [34], [35]. Taken together, it appears that rapid equilibration of osmotic gradients created from the intracellular accumulation of metabolites may be higher with CON-BFR compared with ECC-BFR, which may cause greater muscle cell swelling with CON-BFR.

Our findings showed that the CON-BFR exercise-induced increases in muscle activation may be one of the most important factors for the muscle hypertrophy following CON-BFR resistance training. Recently, Abe et al. [11] reported that exercise intensity estimated from muscle activation is an important factor for BFR training-induced muscle hypertrophy, and the minimum exercise intensity for muscle hypertrophy may be approximately 10% of maximal strength. In the present study, CON-BFR was performed at 30% 1-RM, while the exercise intensity of ECC-BFR was approximately 10% 1-RM (estimated by using the equations of Dalton *et al.* [31]). Therefore, the present and previous findings suggested that the magnitude of muscle activation as well as muscle cell swelling may not be large enough during ECC-BFR exercise to induce muscle hypertrophy.

It should be noted that a potential mechanism for muscle hypertrophy with eccentric training might differ between high-intensity training and low-intensity BFR training. Many studies have compared the effects of resistance training with concentric and eccentric actions, and a systematic review with meta-analysis indicated that eccentric training is superior for increasing muscle size compared with repetition matched concentric training [36]. Compared with concentric actions, eccentric actions are often characterized by reversed motor unit activation, which induces the selective recruitment of the FT muscle fibers [37]. Therefore, eccentric training is more effective than repetition matched concentric training for muscle hypertrophy at the same intensities. In the present study, however, ECC-BFR training was performed at a very low intensity, approximately 10% 1-RM (the exercise intensity was calculated using the equations of Dalton *et al*. [32]), and muscle activation during exercise was not increased, unlike CON-BFR. Thus, this suggests that the stimulus during eccentric muscle actions was not enough to produce muscle hypertrophy. In addition, significant increases in muscle CSA were found at 10 cm above the elbow joint, but not at the mid-upper arm with ECC-BFR. Interestingly, the lower portion of the elbow flexor muscle has been observed to result in greater eccentric exercise-induced muscle damage compared to the mid-upper arm [38]. Therefore, the ECC-BFR induced muscle hypertrophy may be associated with a larger stress in the elbow flexor (10 cm above elbow joint) during eccentric exercise although degree of the stress was low.

In conclusion, our results in the present study suggest that low-intensity concentric BFR training may lead to pronounced muscle cell swelling, the response to which may be an important factor for promoting muscle hypertrophy.
